# Supplementary material for: Developmental Differences on the Internal Reproductive Systems between the Prediapause and Prereproductive Riptortus pedestris Adults
Source: Insects. 2020 Jun 4;11(6):347. doi: 10.3390/insects11060347 (PMC7349029; doi:10.3390/insects11060347)
Supplement: Supplementary file 1 [file insects-11-00347-s001.zip › 20200505-supplimentary information.doc]

**Supplementary information**

**Developmental differences on the internal reproductive systems between the pre-diapause and pre-reproductive *Riptortus pedestris* adults**

**Abdul Hafeez, Bei Li, Wen Liu, Muhammad Nauman Atiq, Xiao-Ping Wang***

Correspondence: xpwang@mail.hzau.edu.cn

**Procedure for measuring reproductive organs**

We measured the different parts of reproductive organs in both male and female *R. pedestris* using scopephoto3.1software as presented below.

**Figure S1.**Marked areas were measured for calibration of different parts in reproductive organ of *R. pedestris* female as length of vitellarium and diameter of lateral oviduct.

**Figure S2.** Marked areas were measured for calibration of different parts in reproductive organ of *R. pedestris* male as length and width of testis, diameter of ejaculatory duct and size of accessory gland.
